# Tuberculome de Bouchut dans la tuberculose multi focale: à propos de quatre cas

**DOI:** 10.11604/pamj.2014.18.326.5214

**Published:** 2014-08-25

**Authors:** Hicham Janah, Ahmed Alami, Hicham Souhi, Adil Zegmout, Hicham Naji-Amrani, Mohamed Raoufi, Hanane Elouazzani, Ismail Abderrahmani Rhorfi, Ahmed Abid

**Affiliations:** 1 Service de Pneumologie, Hôpital Militaire d'Instruction Mohammed V, Rabat, Maroc; 2 Service d'Ophtalmologie, Hôpital Militaire d'Instruction Mohammed V, Rabat, Maroc

**Keywords:** Tuberculome de Bouchut, tuberculose multifocale, fluorescéine, Bouchut tubercles, multifocal tuberculosis, fluorescein

## Abstract

La tuberculose multifocale a connu un regain de fréquence avec la pandémie du SIDA, elle s'observe encore chez des sujets non infectés par le VIH surtout dans les pays en voie de développement notamment au Maroc. Nous rapportons quatre observations de tuberculose multifocale chez trois patients immunocompétents et un patient immunodéprimé. Quatre patients ont bénéficié d'un bilan phtisiologique, biologique, sérologique(HIV), radiologique et d'angiographie à la fluorescéine pour suspicion de tuberculose multifocale. Il s'agit de trois hommes et une femme, d’âge moyen de 44 ans, trois patients sont immunocompétents et un patient séropositif. La tuberculose intéressait trois localisations chez les quatre patients: pulmonaire dans quatre cas, ophtalmique dans quatre cas, digestive dans un cas, urinaire dans un cas, cérébrale dans un cas et un cas d'atteinte de la moelle osseuse. L'atteinte ophtalmologique est représentée par des nodules choroïdiens de Bouchut dans quatre cas et un nodule papillaire de Bouchut dans un cas; aucun des ces patients ne présentait une uvéite granulomateuse. Nos malades ont reçu un traitement anti-tuberculeux d'une durée de neuf mois avec une bonne évolution clinique, biologique, radiologique et angiographique. Au Maroc, la tuberculose continue à surprendre aussi bien par son extension touchant le sujet débilité et le sujet immunocompétent, que par ses présentations diverses y compris l'atteinte oculaire qu'elle faut rechercher par un examen ophtalmologique soigneux et systématique.

## Introduction

La tuberculose est une infection grave. Elle sévit à l’état endémique dans les pays en voie de développement. Sa recrudescence avec l'avènement du SIDA et l'apparition de souches multi résistantes font actuellement de cette maladie un problème de santé publique dans les pays développés. Les atteintes ophtalmologiques liées à la tuberculose sont diverses parmi lesquelles les tubercules de Bouchut. Nous rapportons quatre observations de tuberculose multifocale avec atteinte ophtalmique.

## Méthodes

Quatre patients ont bénéficié d'un bilan phtisiologique, biologique, sérologique(HIV), radiologique et d'angiographie à la fluorescéine pour suspicion de tuberculose multifocale.

## Résultats

**Population à l’étude:** Il s'agit de trois hommes et une femme admis pour une tuberculose multifocale ayant un âge moyen de 44 ans (28 à 78 ans).

**Antécédents:** Le tabagisme était retrouvé chez 2 cas. La sérologie VIH était positive chez un cas.

**Figure 1:**
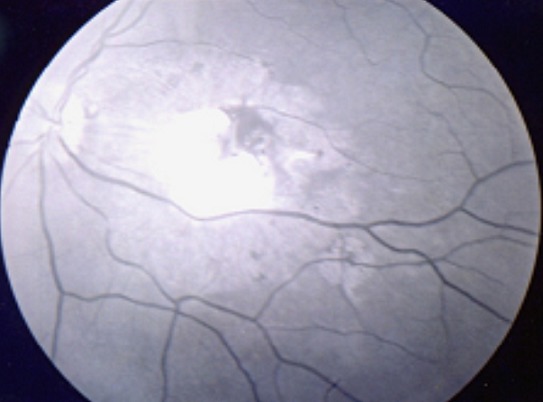
Cliché anérythre de l’œil gauche montrant le tuberculome maculaire

**Figure 2:**
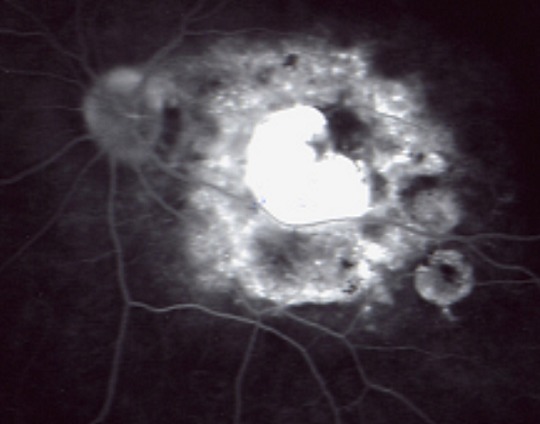
Cliché après injection montrant des foyers de choroïdite périlésionnels, des tubercules de Bouchut, ainsi qu'un œdème papillaire sectoriel

**Figure 3:**
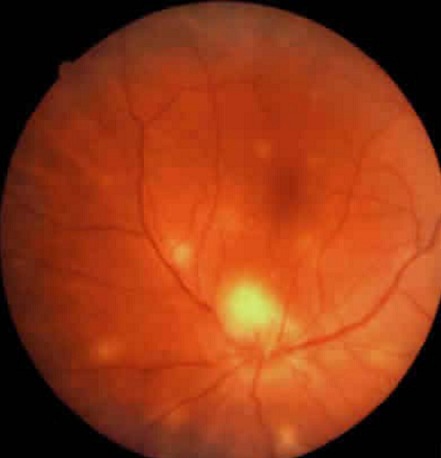
Cliché anérythre de l’œil montrant des nodules blanchâtres sous rétiniens disséminés: tubercules choroïdiens de Bouchut

**Présentation clinique et organes atteints:** Le délai de diagnostic avait une médiane de 60 jours avec quartile de (30,80j). La tuberculose intéressait trois localisations chez les quatre patients: pulmonaire dans quatre cas, ophtalmique dans quatre cas, digestive dans un cas, urinaire dans un cas, cérébrale dans un cas et un cas d'atteinte de la moelle osseuse. L'atteinte ophtalmologique est représentée par des nodules choroïdiens de BOUCHUT dans quatre cas (Figure 1, Figure 2) et un nodule papillaire de BOUCHUT dans un cas (Figure 3); aucun des ces patients ne présentait une uvéite granulomateuse.

**Diagnostic positif:** La tuberculose a été confirmée par recherche de BK dans les crachats chez 2 cas, par étude anatomopathologique chez les autres cas.

**Traitement et évolution:** Tous les patients ont reçu un traitement antituberculeux quadruple à base d'isoniazide, rifampicine, pyrazina¬mide et streptomycine chez 3 cas la streptomycine est remplacée par l'ethambutol chez le séropositif. La duré du traitement était 9 mois.

## Discussion

La tuberculose est une cause majeure de morbi-mortalité dans les pays en voie de développement. La prévalence de la tuberculose dans le monde est estimée à environ 10 millions de nouveaux cas par an. Maroc on recense environ 25 milles nouveaux cas / an [1, 2]. Les localisations extra-pulmonaires représentent 35% de l'ensemble des atteintes, dont 1 à 2% sont des atteintes oculaires [3, 4]. Les atteintes ophtalmologiques liées à la tuberculose sont diverses: toutes les structures de l’œil, à l'exception du cristallin, peuvent être concernées. Certaines de ces manifestations, comme les tuberculomes ou les tubercules de Bouchut, sont liées à la présence du bacille dans l’œil; D'autres, parmi lesquelles certaines formes d'uvéites, sont le reflet d'une hypersensibilité retardée au bacille de Koch [4].

La présence de tubercules de Bouchut d’âge variable, et dont certains sont juxtapapillaires. La découverte fortuite de ces tubercules de Bouchut n'est pas rare, survenant dans 13 à 18% des cas selon les séries. Dans une série prospective de 100 patients atteints de tuberculose, 18 patients avaient une atteinte oculaire, dont 17 une atteinte choroïdienne [5].

Ces observations soulignent la valeur diagnostique des rétinographies en couleurs en tant qu'examen complémentaire de l'angiographie pour la mise en évidence des foyers inflammatoires du vitré postérieurs, difficilement analysés par l'IRM [6]. En revanche, le scanner et surtout l'IRM ont été performants pour l'analyse de la névrite optique. La difficulté du diagnostic bactériologique d'une tuberculose quelle que soit sa localisation, et notamment au niveau intraoculaire, est une réalité incontournable à ce jour [7].

Le traitement antituberculeux standard est bien codifié par des recommandations récentes et débute par une quadrithérapie d'induction associant isoniazide, rifampicine, éthambutol et pyrazinamide pendant 2 mois. Une bithérapie de consolidation associant isoniazide et rifampicine y fait suite pour laquelle une durée de 4 mois semble habituellement suffisante bien qu'une durée de 7 à 10mois est souvent recommandée par précaution. Plusieurs essais cliniques montrent que la corticothérapie adjuvante doit être systématique pour 8 semaines dont 4 à fortes doses [8]. Un traitement symptomatique spécifique doit être mis en place en cas de complications générales, notamment d'hyponatrémie. La surveillance de la tolérance et de l'efficacité du traitement est essentiellement clinique.

## Conclusion

Au Maroc, la tuberculose continue à surprendre aussi bien par son extension touchant le sujet débilité et le sujet immunocompétent, que par ses présentations diverses y compris l'atteinte oculaire qu'elle faut rechercher par un examen ophtalmologique soigneux et systématique.
